# Dual-color augmented reality waveguide display for color vision assistance using color tracking

**DOI:** 10.1016/j.isci.2026.116392

**Published:** 2026-06-12

**Authors:** Seong-Hyeon Cho, Do-Hun Baek, Woo June Choi, Young-Wan Choi

**Affiliations:** 1 Department of Intelligent Semiconductor Engineering, Chung-Ang University, Seoul 06974, Republic of Korea; 2 School of Electrical and Electronic Engineering, Chung-Ang University, Seoul 06974, Republic of Korea

**Keywords:** Optics, Optical imaging, Biophysics

## Abstract

Color vision deficiency (CVD) impairs the discrimination of specific color pairs, particularly red and green, and curative treatments remain unavailable. Existing assistive approaches, such as tinted glasses, contact lenses, and screen-based recoloring methods, can improve color discrimination, but these approaches either modify the entire visual field globally or are constrained to fixed display environments. Here, we propose an augmented reality (AR) display that captures real-time real-world scenes, identifies regions of perceptual difficulty, and superimposes virtual content to assist users with CVD by integrating artificial neural network-based color tracking. The AR system incorporates a fabricated holographic optical element (HOE) integrated into compact waveguide optics, functioning as a dual-color narrowband coupler within a transparent combiner. Optical measurements confirm selective wavelength coupling, and proof-of-concept demonstrations show spatially selective overlay on the target objects. These results suggest the feasibility of HOE-based AR color vision assistance as a wearable approach for targeted color-discrimination scenarios.

## Highlights

• A holographic optical element selectively diffracts two narrow color bands
• A dual-color AR waveguide display assists color discrimination for CVD
• An artificial neural network identifies color-confusion regions in real-time scenes
• Proof-of-concept overlays color cues onto red-green ambiguous targets

## Graphical abstract

**Figure:**
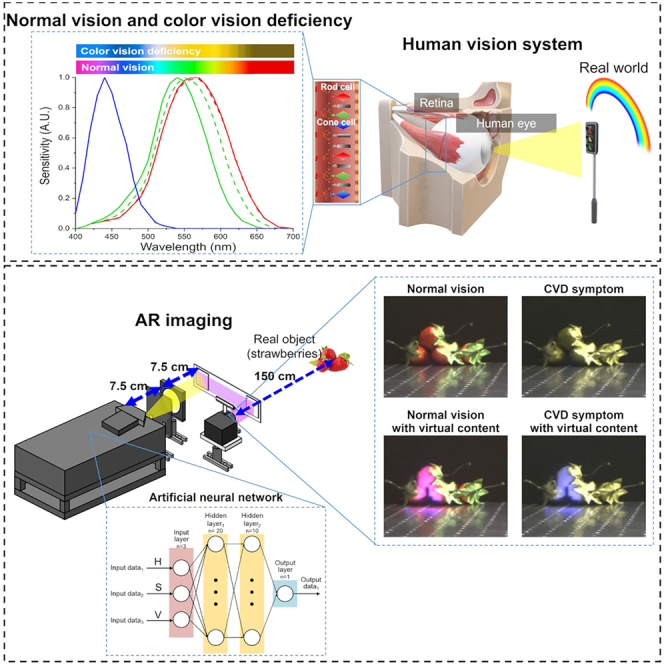

## Introduction

Human vision is closely influenced by cognitive processing, with this influence accounting for more than 80% of sensory information processing. Hence, visual cues exert a direct influence on human perception and behavior.^1^^,^^2^ The visual system detects environmental cues by processing light entering the eye, with the human perceptible visible spectrum spanning 380 to 750 nm.^3^ Incident light on the retina is absorbed by photoreceptors and converted into neural signals, which are then interpreted in the brain to facilitate color perception.^4^ Photoreceptor cells in the retina, including rods and cones, function as light-sensitive neurons. Cone cells, which are active primarily in photopic conditions (i.e., bright environments), are categorized into three types: short-wavelength-sensitive (S cones, peak absorption ∼420 nm for blue (B)), medium-wavelength-sensitive (M cones, ∼530 nm for green (G)), and long-wavelength-sensitive cone(L cones, ∼560 nm for red (R)).^5^ Normal trichromatic vision is enabled through the overlap of these absorption spectra when stimulation of all three cone types is intact. However, functional alterations in S, M, and L cones, which are caused by congenital genetic mutations or acquired factors such as diseases or injuries, result in altered spectral sensitivity. These alterations are collectively known as color vision deficiencies (CVDs).^6^ CVDs affect approximately 3%–4% of the global population, with a higher prevalence in males (approximately 8%) compared to females (0.5%) due to X-linked recessive inheritance.^7^ Individuals diagnosed with CVDs frequently encounter difficulties in discriminating specific color hues, most commonly R and G, and less frequently B and yellow. Such deficiencies have the potential to impede performance in color-critical tasks, thereby imposing occupational limitations in professions such as aviation piloting, firefighting, law enforcement, railway engineering, and associated domains. Although extensive research has been conducted, CVDs primarily arise from genetic factors, and no curative treatments are currently available.^8^^,^^9^^,^^10^^,^^11^ Nevertheless, ongoing efforts have emphasized assistive technologies to support color perception in affected individuals.

These efforts primarily involve correcting indistinguishable hues to enhance color differentiation, enabling classification based on the severity of deficiency. Recently, color-assisting algorithms, mobile applications, and commercial filter-based products have been extensively investigated for supporting individuals with CVD.^12^^,^^13^^,^^14^^,^^15^^,^^16^^,^^17^^,^^18^^,^^19^^,^^20^^,^^21^^,^^22^ Importantly, since CVDs stem from cone cell anomalies, it is challenging to provide CVD patients with the same color perception as those with normal vision. Image processing and recoloring algorithms improve color discrimination by separating visually confusing hues and presenting modified visual information for users.^12^^,^^13^^,^^14^^,^^15^^,^^16^ Mobile-based applications often incorporate diagnostic functions, such as the Ishihara test, and provide personalized interface recoloring based on the user’s deficiency type. Although these approaches can be effective for specific tasks, they are generally limited to fixed display environments such as monitors or smartphones and therefore restrict mobility in real-world scenarios.

Thus, there is a clear demand for smart assistive technologies that function seamlessly in real-time without disrupting routine activities. In response to these requirements, filter-based commercial products, primarily wearable tinted glasses and contact lenses, have been developed.^17^^,^^18^^,^^19^^,^^20^^,^^21^^,^^22^^,^^23^^,^^24^^,^^25^^,^^26^^,^^27^ In 1837, Seebeck introduced color-correcting lenses using filters, and Maxwell subsequently created the first tinted glasses based on this concept. These early innovations allowed CVD individuals using R-G filters to discern previously indistinguishable colors. In recent developments, color discrimination has been enhanced through multi-notch filters that selectively attenuate wavelengths in the overlapping regions of photopigment absorption spectra, as exemplified by eyeglasses such as EnChroma and VINO, and contact lenses such as ColorMax and ChromaGen.^17^^,^^18^^,^^19^^,^^20^^,^^21^^,^^22^ These optics alleviate CVD symptoms by increasing chroma differences for specific colors via multi-notch filtering at the optical surface. Ideally, absorption in targeted wavelength bands should be tailored to the patient’s CVD severity. Furthermore, to optimize the absorption spectra of these filter-based products, investigations have incorporated fluorescent dyes such as Atto 488 and 565, rhodamine derivatives, and silver-gold nanoparticles.^28^^,^^29^^,^^30^ However, these materials often have relatively broad absorption spectra, limiting their specificity to CVD types. Additionally, as external light passes through the filter before reaching the eye, unintended alterations in light perception can occur, potentially diminishing recognition of certain colors and inducing confusion in standard color tasks.^31^^,^^32^^,^^33^ As a result, many studies on tinted glasses and contact lenses report challenges in everyday environmental applications, and the visible tinting may compromise patient privacy. Despite these limitations, such methods are reported to enhance color discrimination in targeted scenarios.

Nowadays, optical see-through (OST) near-eye augmented reality (AR) displays integrated with adaptive optics have emerged as promising assistive devices.^34^^,^^35^ Unlike tinted glasses or contact lenses, OST-AR displays preserve direct real-world viewing while selectively superimposing virtual information onto physical objects or locations, enabling real-time assistance in daily environments.^36^^,^^37^^,^^38^^,^^39^^,^^40^^,^^41^^,^^42^ Head-mounted displays (HMDs) equipped with AR capabilities, which benefit from advances in microprocessors, communications, displays, and optical combiners, are rapidly being developed into compact, smart glasses-like forms. These head-worn AR smart glasses enable hands-free operation, offering substantial potential for entertainment, education, training, and vision assistance, including optical character recognition, object-based visual support, symptom management, and rehabilitation training. OST-type AR displays project images from a light source onto the user’s eye via an optical combiner, facilitating undistorted integration of real-world vision with overlaid content. Optical combiners in these displays incorporate transparent elements, such as macro-optical beam splitters, freeform prisms, or micro- and nano-optic waveguide structures.

Among these, waveguide-based displays utilize thin diffractive optical elements (DOEs) to guide light to the human eye, enabling compact, lightweight designs that prioritize user comfort and making them the most actively researched type of AR display.^40^^,^^41^ DOEs are commonly realized as gratings or holograms, serving as input and output couplers in waveguide displays. They are classified into surface-relief, volume, polarization, and meta-lens types, diffracting light in designed wavelength ranges while transmitting others. Volume optical gratings, fabricated from photosensitive materials by exposing them to two coherent laser beams, are termed holographic optical elements (HOEs).^42^^,^^43^^,^^44^ HOEs provide key advantages, including low fabrication costs and straightforward implementation, as they avoid reliance on semiconductor processes. The resulting 3D periodic refractive index modulation achieves high diffraction efficiency at specific angles and wavelengths. Furthermore, their volumetric structure offers superior angular and chromatic selectivity compared to surface-relief gratings.^44^ Typically, HOEs are implemented in photopolymers and are configured as transmission or reflection types based on the recording beam orientation. Their transmission and reflection spectra depend on the recording wavelength, interference pattern, exposure time, and intensity. HOEs offer high diffraction efficiency and angular and chromatic selectivity under Bragg-matched conditions, making them well suited for narrowband waveguide coupling applications. Multicolor HOEs are fabricated by adjusting diffraction efficiency through exposure parameters and recording multi-wavelength interference patterns.

Based on these advantages, we propose the OST near-eye AR display for color vision assistance in individuals with CVD, incorporating a dual-color narrowband color manipulation-HOE (CM-HOE) for wavelength-selective modulation. Rather than attempting complete optical correction of CVD, this work aims to provide assistance-oriented enhancement of color discrimination under selected viewing conditions. In R-G CVD, R objects often appear visually similar to surrounding G regions due to overlapping responses between M- and L-cone cells. The proposed optics improves practical color discrimination by selectively identifying color-ambiguous regions and providing distinguishable AR cues. This approach enables both object recognition and enhanced color discrimination more effectively than a single-color marker, which would only indicate object location without preserving contextual color information. Unlike non-AR methods that rely on natural light sources, our OST near-eye AR approach preserves the integrity of natural light. Since individuals with CVD experience abnormal color and luminance perception compared to those with normal vision, complete normalization remains challenging. Therefore, we employ the CM-HOE to modulate luminance differences in colors that are difficult to distinguish, thereby assisting color separation within the patient’s visual field. The key distinction from commercialized AR displays lies in the selective coupling of specific wavelength bands from the light source, such as a beam projector or micro display, followed by delivery to the human eye. Since these conventional light sources emit across a wide wavelength band, improvement in CVD symptoms is difficult to expect without wavelength-selective modulation. In contrast, the CM-HOE in this study is realized via customized holographic recording techniques, which selectively diffract two narrow Bragg-matched bands centered near the recording wavelengths into the waveguide while delivering the remaining visible spectrum, thereby preserving see-through perception of the real world.

For this purpose, we introduce a reflection-type dual-color HOE, referred to as CM-HOE, as a narrowband input and output coupler integrated into a compact waveguide display. The CM-HOE selectively diffracts Bragg-matched R and B wavelength bands into and out of the waveguide, delivering spatially registered AR overlay cues while maintaining OST functionality. This study focuses on R-G CVD and combines the dual-color CM-HOE waveguide display with a real-time color tracking module that detects R target regions and overlays corresponding R-B visual cues through a beam projector. We present optical characterization of the fabricated system, including spectral measurements at the waveguide input and eyebox, together with proof-of-concept demonstrations of selective AR overlay on target objects. The experimental results support the feasibility of the proposed system as a wearable platform for improving practical color discrimination in individuals with CVD.

## Results

### Consequences of visual impairment symptoms

**Figure 1:**
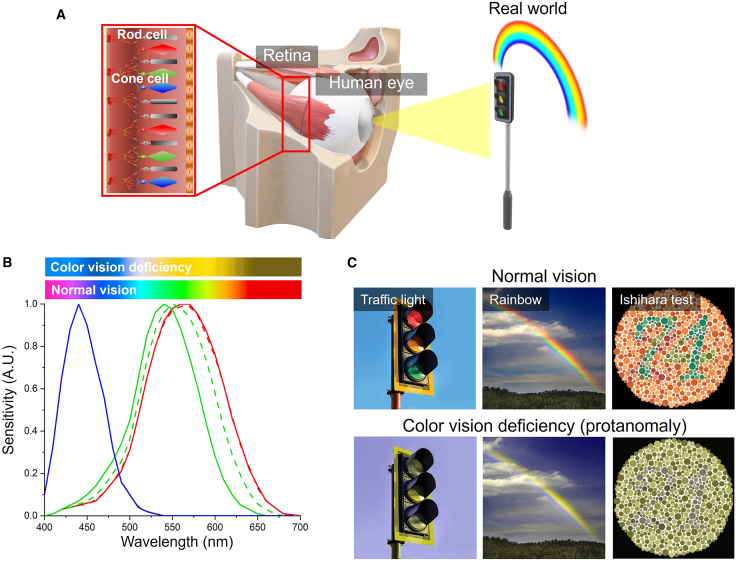
Comparison of visual perception with normal vision and CVD symptom (A) Schematic of the human vision system’s perception of color in the real world. (B) Plot of cone cell sensitivity spectra in normal vision (solid line) and CVD symptoms (dotted line). (C) Image recognition simulation results for normal vision and CVD symptoms.

Natural light incident on the retina triggers wavelength- and intensity-dependent detection, initiating chemical reactions in photoreceptors that convert it into electrical signals for perception, as illustrated in Figure 1A. Rod cells detect brightness and motion, whereas cone cells enable color discrimination. Normal vision relies on the proper functioning of L-, M-, and S-cone cells, which support trichromatic color perception, commonly diagnosed as trichromacy. Depending on the cone cell type and severity, CVDs are classified as anomalous trichromacy, dichromacy, or monochromacy. The most prevalent forms are protanomaly or deuteranomaly, where cone function is diminished. These progress to protanopia or deuteranopia with complete functional loss as severity increases. Individuals with these conditions struggle to distinguish R and G, often perceiving them as similar yellowish tones. Figure 1B compares the response spectra between normal vision and R-G CVD patients, revealing a shifted response peak in patients due to molecular variations in cone cells. This peak shift leads to overlap in the R and G response spectra, thereby hindering color discrimination. Researchers have proposed the LMS color space to visualize perceptual vision for CVD patients on displays.^14^ The LMS space represents sensitivity curves of the three cone types, with peak absorptance in the long-, medium-, and short-wavelength regions of the visible spectrum. These simulations transform trichromacy to dichromacy by incorporating the action spectra of the three cone pigments, reproducing color confusions experienced by dichromats. Conversion from the RGB color space, which is commonly used for displays, to the LMS color space is expressed as in Equation 1:

(Equation 1) $$M_{RGB\to LMS}=\left[\begin{array}{c} L\\ M\\ S \end{array}\right]=\left[\begin{array}{ccc} 17.8824 & 43.5161 & 4.11935\\ 3.45565 & 27.1554 & 3.86714\\ 0.0299566 & 0.184309 & 1.46709 \end{array}\right]\left[\begin{array}{c} R\\ G\\ B \end{array}\right]$$

The Viénot method was developed to assess color legibility from the perspective of CVD symptoms. This approach employs a projection matrix that substitutes the response of the deficient cone type with a linear combination of the remaining two cone types, depending on the CVD variant. This effectively reduces trichromatic colors to a two-dimensional space, simulating the color gamut perceived by individuals with CVD. For protanopia simulation, which is the most prevalent form of dichromacy, the representation in LMS space includes additional linear transformations that apply the protanomaly matrix values as in Equation 2:

(Equation 2) $$M_{protanomaly}={\left[\begin{array}{c} L^{\prime }\\ M^{\prime }\\ S^{\prime } \end{array}\right]}_{protanomaly}=\left[\begin{array}{ccc} 0 & 2.02344 & -2.52581\\ 0 & 1 & 0\\ 0 & 0 & 1 \end{array}\right]\left[\begin{array}{c} L\\ M\\ S \end{array}\right]$$

Figure 1C displays the original images (traffic lights, rainbow, and Ishihara test) alongside protanomaly simulation results, which integrate the linear transformation in LMS space to depict how these images appear in the visual field of patients with CVD. Individuals with R-G CVD perceive real-world objects as indistinct, yellowish tones without clear R-G differentiation. Notably, the Ishihara test, used as a diagnostic tool for color deficiencies, shows inadequate color contrast under CVD conditions, making pattern recognition difficult. Such perceptual challenges increase accident risks due to color confusion in traffic signals and food products. Therefore, improving color contrast according to the level of R-G distinction can support color discrimination in affected individuals.

### Fabrication of dual-color-based optics

**Figure 2:**
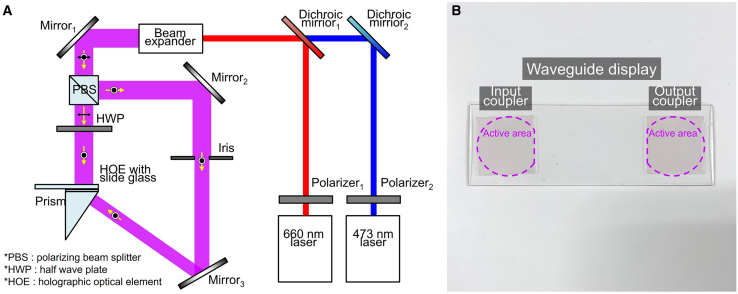
Schematic of an experiment for CM-HOE manufacturing and an implemented CM-HOE-based waveguide display (A) Interferometer for CM-HOE fabrication using laser beam sources at 473 and 660 nm, with applied exposure time and intensity for each beam source. (B) CM-HOE-based waveguide display with the active areas recorded within the interference pattern of 473 and 660 nm laser beams.

In previous studies, it has been recommended to selectively attenuate wavelength bands in the ranges of 480–500 nm and 550–580 nm to assist color vision in patients with CVD.^21^^,^^22^ The 480–500 nm region represents the intersection between S and M cone responses (G-B crossover), leading to confusion between cyan and G hues, whereas the 550–580 nm region corresponds to the intersection between M- and L-cone responses (R-G crossover). The mechanism underlying these literature recommendations is to reduce stimulation of the M and L cones in their overlapping response region, thereby enhancing the relative contrast between independent cone signals. Inspired by this principle but adopting a fundamentally different optical strategy, our optics achieves the same goal in an additive manner. Instead of attenuating light from the natural scene, we add narrowband AR cues at wavelengths that selectively stimulate the S and L cones while bypassing the cone-crossover regions altogether. Specifically, the CM-HOE is recorded with diode-pumped lasers at 473 and 660 nm to selectively diffract two narrow Bragg-matched bands into the waveguide as AR overlay cues, while the remaining visible spectrum, which includes the cone-crossover regions, is transmitted unaltered via the see-through path. Based on the proposed AR assistance strategy for CVD, a dual-color reflection-type CM-HOE was therefore designed and fabricated as the waveguide display coupler. The experimental setup for implementing the CM-HOE is illustrated in Figure 2A. The light sources at 473 and 660 nm were reflected by respective dichroic mirrors and combined to overlap during propagation. The propagated beams were then expanded through the 20× beam expander. The expanded light was reflected at 90° by a mirror and separated into S- and P-polarized components via a 50:50 polarizing beam splitter, with the S-polarized light reflected and the P-polarized light transmitted. The S-polarized light was directed at 60° by an arranged mirror and entered vertically into a 60° triangular prism, while the P-polarized light was converted to S-polarization by a half-wave plate and transmitted. The propagated coherent S-polarized lights were precisely aligned and interfered within a 15 × 15 mm size of photopolymer, serving as the recording medium. This interference pattern induces the chaining of the irregularly aligned photo-initiators and monomers within the photopolymer, forming periodic patterns and implementing refractive index modulation. Additionally, holographically fabricated grating reconstructs the diffracted beam by propagating a beam of identical conditions along the alignment of the recording beam. The photopolymer was attached to a transparent flat slide glass, functioning as the waveguide substrate, and was closely affixed to the glass surface of the established 60° triangular prism. The air gap between the prism and slide glass was eliminated by uniformly applying glycerin with a refractive index of 1.44 for index matching. The intensity of the signal and reference beam separated from each beam source was strictly the same to ensure the interference condition. The beam at 473 nm was exposed for 10 s at a fixed intensity of 3 mW/cm^2^ to achieve an energy level of 30 mJ/cm^2^. Conversely, the 660 nm beam was applied sequentially at exposure intensities ranging from 0.5 to 1 mW/cm^2^ in 0.5 mW/cm^2^ steps until a total energy of 30 mJ/cm^2^ was reached. The beam at 473 nm was then subjected to an exposure intensity adjustment in order to acquire six types of CM-HOE samples. The fabricated samples were then utilized to measure the diffraction efficiency in the R and B wavelength bands. Glycerin was completely removed to ensure the quality of the waveguide display, and the fabricated CM-HOE was cured under UV light for 3 min to prevent additional deformation from external light exposure. By repeating the process, the CM-HOE-based waveguide display was completed by attaching photopolymer film-based input and output couplers to both ends of the slide glass. The fabricated CM-HOE-based waveguide display was realized as a transparent optical combiner, as shown in Figure 2B.

### Measurement and evaluation of CM-HOE’s diffraction efficiency

**Figure 3:**
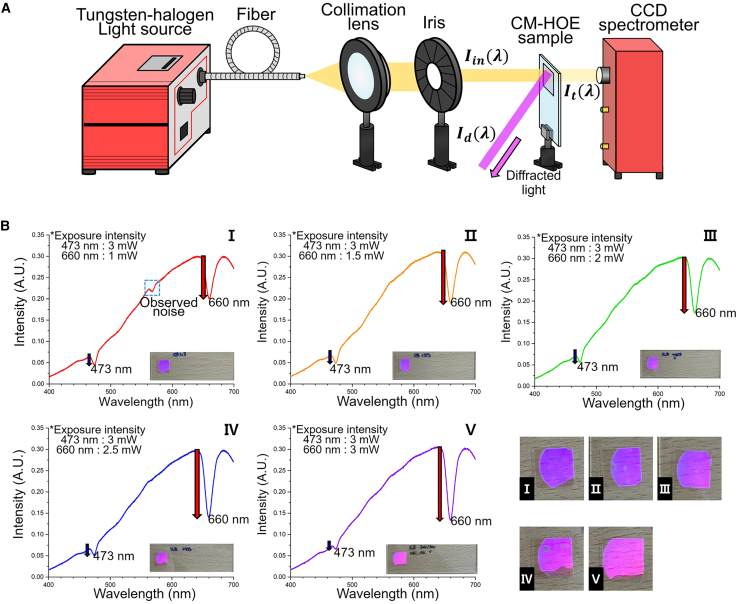
Experimental process for measuring the transmission spectrum of CM-HOE samples (A) Schematic of optical measurement system using a tungsten-halogen light source and a CCD spectrometer. (B) Transmission spectra of CM-HOEs depending on the exposure beam intensity at 473 and 660 nm and a photo of implemented CM-HOE samples.

The diffraction characteristics of the fabricated CM-HOE were experimentally evaluated using transmission spectrum measurements of samples exhibiting different diffraction efficiencies in the R and B wavelength regions. Because diffraction efficiency strongly depends on the exposure conditions and the intrinsic sensitivity of the photopolymer film, CM-HOE samples were fabricated by adjusting the exposure intensity during holographic recording. To characterize the spectral response of the fabricated HOE, the transmission spectrum across the visible range was measured using a fiber-coupled tungsten-halogen light source and a CCD spectrometer. The processed CM-HOE was precisely positioned in the path of the collimated light source from the CCD spectrometer via an arranged lens, as depicted in Figure 3A. Here, *I*_*in*_ denotes the incident intensity, *I*_*d*_ is representative of the diffracted intensity, and *I*_*t*_ signifies the transmitted intensity. Neglecting absorption and reflection losses in the photopolymer and substrate, the diffraction efficiency *η*_*d*_ was calculated using Equations 3 and 4:

(Equation 3) $$I_{d}\left(\lambda \right)=I_{in}\left(\lambda \right)-I_{t}\left(\lambda \right)$$

(Equation 4) $$\eta_{d}\left(\lambda \right)=\frac{I_{d}\left(\lambda \right)}{I_{in}\left(\lambda \right)}\times 100$$

The measured transmission spectrum for each sample is presented in Figure 3B, where the fabricated photopolymer exhibited relative diffraction efficiencies of 11%–40% and 36–57% at central wavelengths of 473 and 660 nm, respectively. These measurement results suggest that the diffraction efficiency in the R and B color regions can be adjusted based on the photopolymer’s inherent characteristics at different exposure intensities. Moreover, the results indicate that the narrow Bragg-matched bands centered at 464–479 nm and 651–682 nm are selectively diffracted by the CM-HOE, whereas all wavelengths outside these two narrow bands, including the wider R, G, and B color regions of the visible spectrum, are primarily transmitted via the photopolymer film without being coupled into the waveguide. Consequently, the CM-HOE functions as a dual-color narrowband coupler that selectively couples the two Bragg-matched bands into the waveguide for delivery to the eye via the waveguide display.

**Figure 4:**
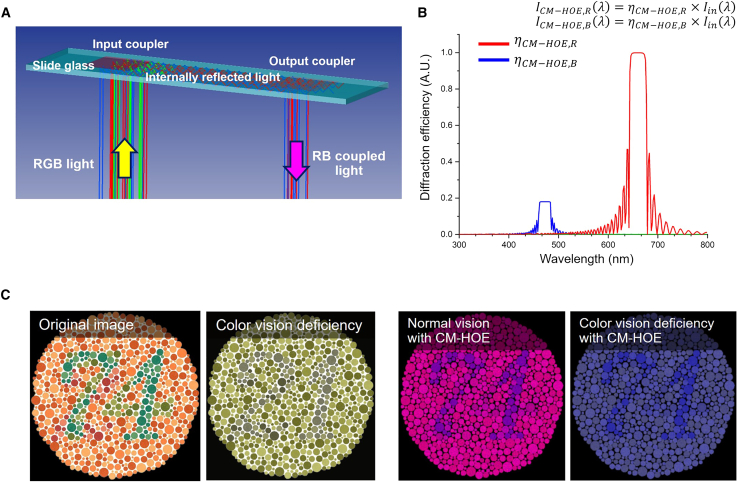
Zemax simulation process and results for theoretical proof-of CM-HOE-based waveguide display (A) Ray tracing setup of RGB beams by CM-HOE couplers with chromatic selectivity. (B) Plot of relative diffraction efficiency versus wavelength for CM-HOE-based on Kogelnik’s theory. (C) Normal and CVD symptoms simulation result with CM-HOE using the Ishihara test target.

Figure 4A illustrates the structure of the CM-HOE-based waveguide display fabricated as depicted in Figure 2B, designed using the optical simulation tool Zemax OpticStudio, which demonstrates the propagation process of coupled light in the proposed optics. To match the Bragg condition, the RGB light source was incident vertically onto the input coupler of the CM-HOE-based waveguide display, using the same incidence geometry as the reference beam applied during holographic recording. Subsequently, the R- and B-wavelength lights were diffracted at 60°, satisfying the total internal reflection (TIR) condition (≈41.8°) and undergoing TIR within the waveguide substrate. The propagated R- and B-wavelength lights were then diffracted again by the output coupler and propagated vertically toward the user’s eye. Additionally, we derived optimized relative spectra in the R and B wavelength regions as a function of exposure intensity and time, as shown in Figure 4B, based on Kogelnik’s theory, which accounts for the wavelength dependence of the spectrum-measured HOE by considering factors such as the thickness, refractive index, incident angle, recording wavelength, index modulation, and shrinkage rate of the photopolymer film used.^45^ This derivation incorporated the response levels in the corresponding wavelength regions from the visual system’s spectrum, along with the HOE’s R and B efficiencies, and was calculated to achieve high color contrast between the two colors. Figure 4C presents simulation results incorporating human response spectra and the calculated spectra, utilizing the Ishihara test target to compare the color contrast between R and G. In normal vision, owing to the relatively high diffraction efficiency for R, R and B appeared as magenta and B hues, respectively. Subsequently, CVD linear matrix values were additionally applied to the calculated normal vision images to represent the visual field as perceived by CVD patients. In the CVD vision, the simulated image was predominantly rendered as yellow color, complicating number discrimination in the image. However, simulations incorporating the CM-HOE produced images in which the number 74 within the displayed image appears more distinguishable. Equation 5 expresses the simulated CVD output by linearly transforming sample images (*I*_*in*_) from RGB space to LMS space, which applies to the human vision system, incorporating the transmission spectrum of the designed holographic coupler, *T(λ)*, along with the CVD linear matrix:

(Equation 5) $$I_{out}=I_{in}\times M_{RGB\to LMS}\times \int_{300nm}^{800nm}T\left(\lambda \right)d\lambda \times {\left[\begin{array}{c} L^{\prime }\\ M^{\prime }\\ S^{\prime } \end{array}\right]}_{protanomaly}$$

The simulated CVD images (*I*_*out*_) with CM-HOE showed that the R and B Bragg-matched bands were selectively coupled into the waveguide. To assist CVD patients in discriminating colors, this selective coupling of R and B in the corresponding regions can increase the color contrast between them. These results provide the optical basis for the subsequent real-time AR overlay system.

**Figure 5:**
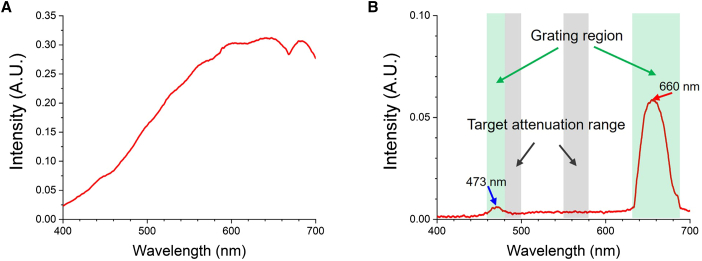
Input and output spectra of the CM-HOE-based waveguide display (A) The spectral range of tungsten-halogen light source at the waveguide input. (B) Measured transmission spectrum at the eyebox of CM-HOE-based waveguide display.

We fabricated the waveguide display using CM-HOEs as input and output couplers, based on the measured spectral response of the CM-HOE sample shown in Figure 3. The spectra of the light incident on the input coupler and of the light delivered to the eyebox by each coupler were measured utilizing the approach illustrated in Figure 3A. The light source employed in the experiment was a tungsten-halogen lamp, whose spectrum is presented in Figure 5A. The tungsten-halogen lamp emits broadband radiation owing to blackbody radiation and is unpolarized, as numerous atoms vibrate randomly in all directions. This light source was collimated by the optics in Figure 3A to satisfy the HOE reconstruction conditions and directed normally toward the CM-HOE-based waveguide display. Upon incidence onto the glass waveguide, the light underwent 60° diffraction at the reflection-type CM-HOE input coupler, where the Bragg-matched light in the recorded R and B wavelength bands was selectively diffracted to the waveguide’s output coupler. This behavior arises from the intrinsic property of the volume holographic grating, which reconstructs and redirects only wavelengths that satisfy the Bragg condition. The light then propagated to the reflection-type CM-HOE output coupler, where it was again diffracted symmetrically at 60° and exited normally. The spectrum measured at the eyebox region, as shown in Figure 5B, indicates the diffraction efficiencies of each CM-HOE for the grating regions in the R and B wavelength bands. Consequently, most off-Bragg wavelengths outside the recorded R and B bands were transmitted unaltered via the see-through path. In contrast, the Bragg-matched R and B wavelengths were selectively diffracted from the direct see-through path. The output intensity at the eyebox was lower than that of the input light due to the coupler’s diffraction efficiencies and optical losses in the glass substrate. Although the B wavelength band intensity at the eyebox appeared relatively low because of the spectral characteristics of the tungsten-halogen source, the relative ratio between the R and B wavelength bands remained consistent with the intended proportion when normalized to the input light source spectrum. As a result, the eyebox output light satisfied the target spectral region of the dual-color narrowband coupler. In AR-HMDs, this light is propagated to the human eye. Additionally, the input light source can be replaced with a beam projector or micro display to overlay color-assistance images onto real-world objects that are difficult for CVD patients to distinguish.

We quantitatively evaluated color discrimination performance for CVD patients using the measured spectrum in Figure 5. This evaluation was performed using the input light spectrum in Figure 5A and eyebox output light spectrum presented in Figure 5B with the CIE 1976 u'v' color space. The CIE 1976 u'v' color space is a coordinate system that transforms the CIE 1931 xy color space into a more perceptually uniform scale, designed to better correspond to human color perception differences.^46^^,^^47^^,^^48^ The *u'* and *v'* coordinates are derived from the *x* and *y* coordinates of the CIE 1931 color space as in Equations 6 and 7:

(Equation 6) $$u^{\prime }=\frac{4x}{-2x+12y+3}$$

(Equation 7) $$v^{\prime }=\frac{9y}{-2x+12y+3}$$

In the CIE 1976 u'v' color space, regions where individuals with CVD have difficulty distinguishing colors are represented by confusion lines.^47^^,^^48^ Colors lying on these lines produce nearly identical stimulation to specific cone cells, making them difficult to discriminate. In general, the color reproduction performance of displays and lighting systems improves as the Δ*u'v'* value decreases, resulting in higher color accuracy and uniformity. In contrast, for individuals with CVD, specific color pairs are perceived as nearly identical when they lie on a confusion line. Therefore, one approach to assist color discrimination in CVD patients is to separate colors by a sufficient perpendicular distance (Δ*u'v'*) from the confusion line.^49^^,^^50^ The perpendicular separation from the confusion line is calculated using Equation 8:

(Equation 8) $$\Delta u^{\prime }v^{\prime }=\frac{\left|\left(v_{i}-v_{p}\right)\left(\;u^{\prime }-u_{p}\right)-\left(u_{i}-u_{p}\right)\left(v^{\prime }-v_{p}\right)\right|}{\sqrt{{\left(u_{i}-u_{p}\right)}^{2}+{\left(v_{i}-v_{p}\right)}^{2}}}$$

Where *u'* and *v'* represent the chromaticity coordinates of the output light measured at the eyebox of the proposed optics. Additionally, *u*_*i*_ and *v*_*i*_ are the chromaticity coordinates of the input light before it passes through the couplers. The values u_p_ = 0.658 and v_p_ = 0.501 correspond to the standard protanopic copunctal point based on the CIE 1931 2° observer.^51^^,^^52^ We quantified the degree of separation from the protanopic confusion line in the CIE 1976 u'v' color space using the Δ*u'v'* metric. Based on the spectra shown in Figures 5A and 5B, the chromaticity of the input light before the couplers was *u*_*i*_ = 0.23798, *v*_*i*_ = 0.52864, while the output light measured at the eyebox after passing through the two couplers was *u'* = 0.31896, *v'* = 0.49616. Substituting these values into the perpendicular separation formula yielded Δ*u'v'* = 0.02709. This value was larger than the shifts typically observed with commercial notch filters such as EnChroma.^53^ The result arises because the CM-HOE selectively modulates the R and B components of the spectrum, inducing a perception bias toward magenta and purple hues, which are farther from the protanopic confusion line. Perpendicular separation can be further increased by shifting the output light chromaticity in the perpendicular direction within the u'v' color space. This separation can be further increased by partially shifting the wavelength band in the R region, which induces a decrease in the *v'* coordinate. Furthermore, since the light emitted by the proposed optics is overlaid on real objects in an AR display, it can provide color discrimination assistance in specific regions for CVD patients.

### Artificial neural networks for color extraction

**Figure 6:**
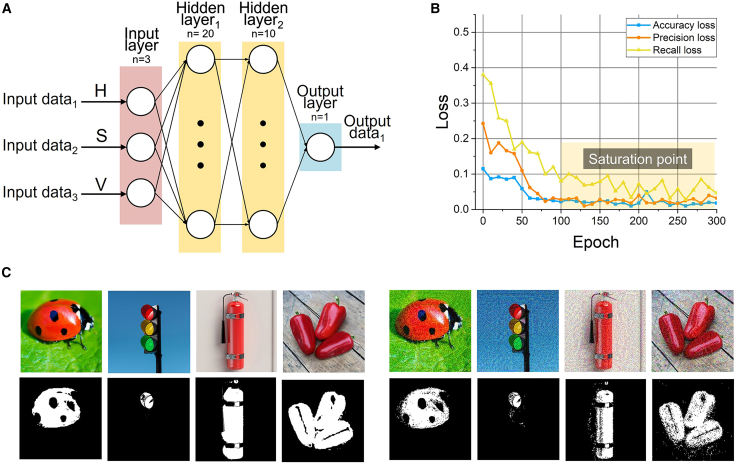
Process and results of HSV-based ANN for color detection and extraction (A) Neural network architecture based on color tracking in the HSV color space. (B) Plot results of accuracy, precision, and recall of the ANN architecture as a function of the number of epochs. (C) Comparison of computational results for original and FGSM (ε = 0.1) simulated image sets.

The RGB color space describes colors based on the intensities of three primary colors (R, G, and B), whereas the HSV (hue, saturation, value) color space expresses colors in terms of hue, saturation, and value. Additionally, the RGB color space presents a challenge in color extraction due to the color distortion caused by brightness variations resulting from the correlation between RGB channels. Conversely, the HSV color space facilitates the extraction of specific color ranges independently, with hue directly representing the fundamental essence of color and value providing illumination invariance.^54^^,^^55^^,^^56^ To focus on this approach, real-time color detection algorithms utilizing adaptive thresholding in the HSV color space are commonly applied to efficiently identify and classify specific colors, even in dynamic outdoor scenes, despite variations in illumination and noise.^57^^,^^58^ Inspired by the advantages of these color extraction algorithms, we introduced a color tracking method in the proposed optical system that overlays virtual content exclusively on regions requiring color discrimination, while allowing unhindered recognition of other colors. We initially targeted the extraction of R object colors and the marking of corresponding regions. The method for color detection and extraction using a tiny camera was implemented in the following sequence. First, input data were converted from the RGB color space to HSV and separated into H, S, and V parameters in the input layer. Subsequently, these were processed through two consecutive hidden layers with weighted computations, yielding a binary output to determine the presence of R. The schematic in Figure 6A illustrates the architecture of the proposed algorithm’s artificial neural network (ANN). The proposed algorithm consists of 20 nodes in the first hidden layer and ten nodes in the second hidden layer. A symmetric sigmoid function was incorporated into the ANN for weight initialization and training. To define the R color range, we utilized an image set of 1,000 images predominantly featuring red objects under varying illumination conditions, such as indoors and outdoors, low light, and strong shadows. Figure 6B plots the loss of accuracy, precision, and recall as a function of the epochs in the proposed ANN architecture. Accuracy measures the proportion of correct predictions among all predictions, precision quantifies the fraction of true positives (TPs) among predicted positives, and recall indicates the fraction of TPs among actual positives. The following equations (Equations 9, 10, and 11) define these parameters:

(Equation 9) $$Accuracy=\frac{TP+TN}{TP+FN+FP+TN}$$

(Equation 10) $$Precision=\frac{TP}{TP+FP}$$

(Equation 11) $$Recall=\frac{TP}{TP+FN}$$

Each parameter was derived from the confusion matrix, categorizing outcomes as TP, true negative, false positive, or false negative. To ensure the reliability of the proposed architecture, the images were split into three groups: training data (70% of the total images), validation data (15%), and test data (15%). We observed that the loss of accuracy, precision, and recall reached saturation point after 100 epochs and stabilized with no further significant loss or fluctuation. Figure 6C presents the original image and the results applying the fast gradient sign attack (FGSM) technique^59^ to demonstrate the robustness of the proposed algorithm. This indicates the potential to extract and track desired colors beyond R through tailored color ranges and training. FGSM technique utilized a gradient of loss to distort the input and is a typical method for model robustness evaluation. The *x’* denotes the perturbed image, *x* the original image, *ε* the intensity, *J* the loss function, and *y* the label of the target, as expressed in Equation 12:

(Equation 12) $$x^{\prime }=x+\epsilon \cdot sign\left(\nabla_{x}J\left(\theta ,x,y\right)\right)$$

Even when an intensity of 0.1 was applied, simulated images produced by FGSM were similar to the original image. From an intensity of 0.2, the image tended to become noisier and more distorted, which was reflected in the masked results. However, since the CM-HOE-based AR display targets relatively large-scale real objects that are difficult for CVD patients to distinguish in color, this was not considered a decisive issue in the current proof-of-concept system.

### Experiments

**Figure 7:**
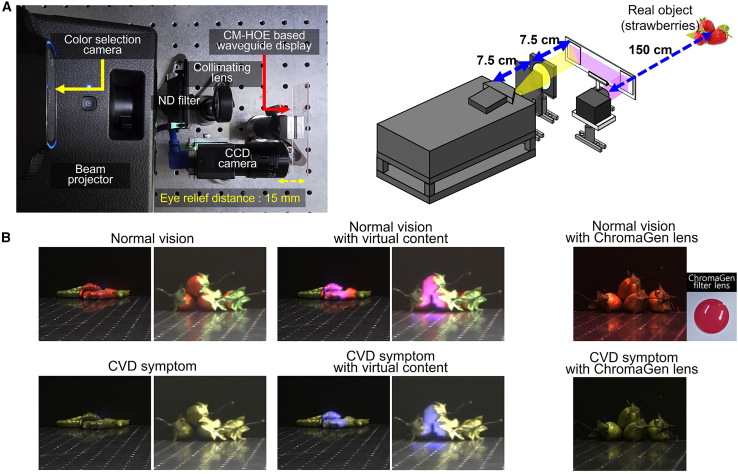
Experimental process for color vision assistance using the proposed near-eye AR display and comparison with a commercial color filter lens (A) Dual-color-based waveguide display for the proof-of-concept color vision assistance. (B) AR display and color filter lens (ChromaGen lens) experimental results with normal vision and CVD symptom simulation.

For the proof-of-concept experiment, the proposed optical system was constructed as illustrated in Figure 7A. The waveguide-based AR display consisted of a beam projector serving as the light source, a convex lens (f = 50 mm) for collimating the light, and the CM-HOE-based waveguide optical combiner. A digital webcam was employed as the camera for color detection from real objects of specific hues and was fixed in alignment with the waveguide-based AR display. The color detection camera identified real object colors in real time, and the segmented regions were transmitted to the beam projector for image output. This camera was also positioned to capture the scene within the acquisition view of the CCD camera. The image output of the beam projector was aligned to accurately deliver the image to the input coupler of the waveguide display. The emitted image was collimated by the arranged lens and incident on the waveguide display, where it was diffracted by the attached input coupler CM-HOE, underwent TIR, and was propagated to the output coupler. Subsequently, the collimated image was diffracted again by the output coupler and vertically incident on the fixed CCD camera. To simulate the typical distance between the eye and eyeglasses, the distance between the CCD camera and the waveguide display’s coupler (eye relief distance) was set to 15 mm.

Figure 7B presents the view captured by the CCD camera using the proposed near-eye AR display, demonstrating the overlay of virtual images onto the color-extracted regions from real objects. The real objects utilized were those that mix R and G hues, which are difficult to distinguish under CVD conditions, such as models of peas, peppers, and strawberries. These real objects were positioned 150 cm from the CCD camera. Figure 7B shows the captured results under normal vision for each object, with virtual image overlays applied on the corresponding observed photos. Under CVD simulations with R anomaly levels, the objects appeared with improved discernibility of color. As simulated in Figure 1C, which represents the CVD symptom view, the RGB channels of the AR image were converted to the LMS color space. The protanomaly matrix was applied in this LMS color space, as previously outlined. The experimental results illustrate the color vision assistance provided by the proposed system. The real world was clearly observed through the transparent CM-HOE-based waveguide display. However, the ChromaGen filter lens showed limited contrast changes for the CVD vision tested under our simulated conditions. While it improved color perception for normal vision and CVD simulations involving real objects with B components, such as magenta and jade, it showed limitations in daily-life conditions. In addition, since the ChromaGen filter lens is placed near the eye when applied to glasses, it cannot selectively deliver assistance to specific areas that require color perception.

The ANN technique for color extraction was processed on a laptop with an Intel i5-10210U processor and 8 GB of RAM. Additionally, the end-to-end latency of our benchtop prototype was measured to range from 38.5 to 52.7 ms. While this latency is currently insufficient for highly dynamic real-time scenes, it is competitive with the processors used in many commercially available AR glasses.

## Discussion

**Figure 8:**
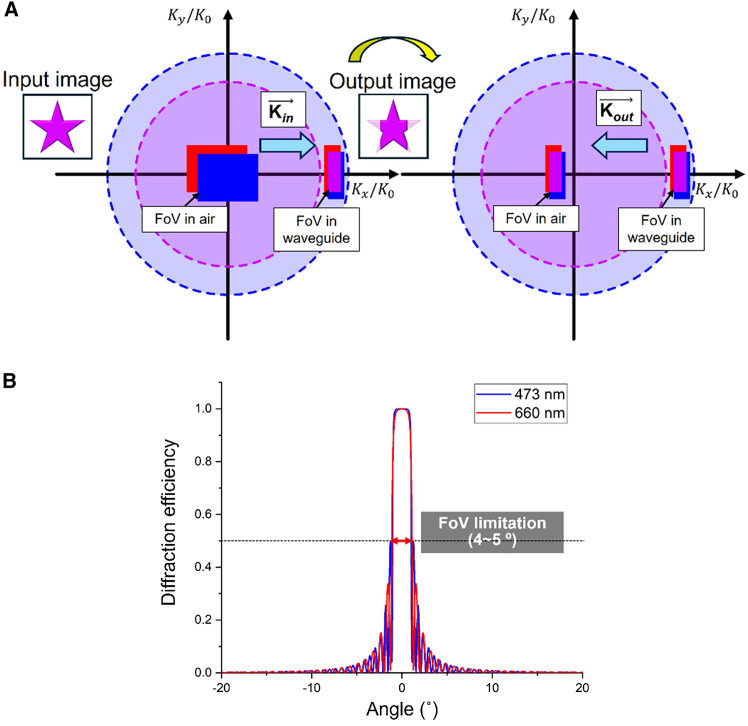
Limitations of FoV due to the image propagation process in the proposed optics (A) Schematic of 2D exit pupil expansion corresponding to the k-vector diagram. (B) Diffraction properties of the proposed reflection-type HOE.

Although the proposed dual-color-based AR waveguide display has demonstrated color vision assistance via the overlay of virtual content, several issues need to be addressed for practical application. First, CM-HOE was recorded using laser beam sources at 473 and 660 nm for proof-of-concept. It is evident that the central diffraction range within the transmission spectrum of CM-HOE depends on the recording beam source. Consequently, two beam sources set lower than the B laser wavelength used and higher than the R laser wavelength can be considered for color vision assistance. Second, holographic film degrades under prolonged external light exposure due to photochemical changes in the photopolymer material, leading to reduced diffraction efficiency over time. The impact includes compromised holographic film quality and lower image resolution. Additionally, the resolution of the proposed display was measured to be 14.2 lp/mm with a modulation transfer function of 0.5, due to the relatively low thickness and refractive index modulation of the photopolymer film used. As an alternative, diffractive grating elements fabricated via semiconductor techniques, such as electron-beam lithography, could provide higher resolution and more stable, light-resistant structures.^60^^,^^61^^,^^62^ Additionally, holographic film can improve the degradation and extend the lifetime of photopolymers by applying encapsulation methods to stabilize crosslinks after recording polymer materials, and 3D printing techniques using panchromatic photopolymer resins.^63^^,^^64^ Future implementations can be explored using holographic volume grating based on hybrid materials to balance durability, potentially extending the diffractive optics lifespan. Third, the fabricated reflection-type holographic coupler primarily diffracts R and B light at different TIR angles due to wavelength differences, potentially causing misalignment at the output coupler and resulting in incomplete image positioning. Figure 8A provides a schematic illustration of these results, where the light escaping from the waveguide is synthesized into magenta. This could degrade AR overlay, especially for dynamic color vision assistance. Mitigation strategies include fine-tuning incident angles per color to synchronize TIR paths or optimizing waveguide and coupler lengths alongside TIR angles.^65^^,^^66^ Advanced simulation and fabrication could further refine these parameters, enabling seamless multicolor integration.^67^ Furthermore, HOEs exhibit narrow angular selectivity (typically ∼4–5° field of view [FoV]) due to constraints in grating thickness and index modulation according to Kogelnik’s coupled-wave theory, as shown in Figure 8B. The proposed optics were also measured with an eyebox size of 42 mm^2^ and a FoV of 4.4°. This limitation arises because the efficiency of diffraction drops sharply outside a small angular range, restricting the CVD patient’s visual field and hindering adequate color vision assistance in dynamic AR scenarios.

Several strategies are expected to be employed to expand the FoV, such as high-refractive-index waveguide substrate and holographic materials, and 2D exit pupil extension (2D-EPE) structures.^68^^,^^69^^,^^70^^,^^71^^,^^72^ The 2D-EPE structure enlarges the exit pupil of the incident light in both horizontal and vertical directions. This provides a wider FoV, a large eyebox, and high brightness uniformity. The 2D-EPE structure addresses the étendue bottleneck and enables the design of a compact AR display. Consequently, these approaches can extend the FoV beyond 20°, potentially enhancing usability for color vision assistance while maintaining compactness.

The proposed CM-HOE was implemented with a relatively broad notch feature (∼10–20 nm bandwidth). This necessitates the optimization of a narrower notch to improve color selectivity in the current proof-of-concept design. The wavelength and angular selectivity in the HOE are determined by the thickness and modulation of the refractive index of the diffraction grating. HOE with narrow bandwidth increases diffraction efficiency at specific wavelengths, but an excessively narrow bandwidth significantly reduces diffraction efficiency in wavelength regions that do not satisfy the Bragg condition. Furthermore, this can cause noise due to increased higher-order diffraction, potentially degrading the quality of the displayed virtual image. Moreover, deviations of the wavelengths and incident beam angles from those of the recording beam degrade overall image quality, as the diffraction efficiency depends sensitively on these parameters. Consequently, angular and chromatic uniformity cannot be guaranteed. Given these requirements, this approach entails a trade-off between notch bandwidth, diffraction efficiency, and color discrimination performance. Therefore, this trade-off relationship should be carefully considered for optimization of the optical performance factors.

The CM-HOE-based waveguide display was recorded through parallel beams of R and B laser light sources. Therefore, the reconstruction of the beams is diffracted in parallel and delivered to a photoreceiver, such as a camera or human eye. As a result, the virtual image is fully visible when the real-world object is placed at a considerable distance from the photoreceiver. However, at relatively close distances, the mismatch between the focal plane of the virtual image and the focus cue of the photoreceiver can cause a vergence accommodation conflict. This mismatch can be mitigated by applying optics such as a holographic lens, which utilizes the intrinsic characteristics of photosensitive materials, or a varifocal-based focus-tunable lens.^73^ Furthermore, although the proposed optics were implemented in a benchtop prototype to demonstrate proof-of-concept, the simple and compact optical system has the potential to be implemented as a smart assistive device for color vision assistance in the form of glasses by using small commercial beam projectors, cameras, and processors. However, the prolonged wear of AR displays remains challenging due to issues with thermal management and power efficiency.^74^

In summary, this study presents a proof-of-concept for a compact optical system that provides color vision assistance through AR near-eye displays. By integrating a customizable HOE with a waveguide-based AR platform, the proposed method offers a potential alternative pathway to traditional CVD aids, such as filter-based tinted glasses and contact lenses, which suffer from natural light distortions and lack of real-time adaptability. The optical system features a simple design incorporating an ANN-based color tracking algorithm that enables real-time detection and segmentation of hard-to-distinguish hues, allowing precise overlay of virtual content in specific regions without altering the entire visual field. This versatility is enabled by the angular and wavelength selectivity of the dual-color narrowband HOE coupler in a transparent optical combiner, ensuring undistorted real-world perception. Furthermore, the optics have the potential to be tailored to varying CVD severities, extending applicability beyond protanomaly and deuteranomaly. Therefore, the system holds potential to support daily visual experiences for CVD patients and can broaden opportunities in color-dependent tasks such as aviation or emergency services. Ultimately, this AR system illustrates a possible direction for smart assistive devices and personalized visual augmentation.

### Limitations of the study

Although the proposed CM-HOE-based AR waveguide display demonstrates the optical feasibility of a narrowband dual-color coupler for CVD assistance, the study has several limitations that should be acknowledged. First, the assistance effect was evaluated via optical measurements, including the chromaticity separation Δ*u′v′* from the protanopic confusion line, and proof-of-concept benchtop demonstrations rather than direct perceptual validation with individuals with CVD. A dedicated user study with CVD-affected participants under real-world viewing conditions would be required to establish the perceptual benefit and quantify the assistive effect in everyday color-discrimination tasks. Second, the FoV (4.4°) and eyebox (42 mm^2^) of the current benchtop prototype are limited by the angular selectivity of the volume holographic grating, restricting applicability in dynamic AR scenarios that require wider visual coverage. Third, the photopolymer-based CM-HOE is susceptible to long-term degradation under prolonged exposure to light, a risk that must be mitigated through encapsulation or alternative diffractive materials to enable sustained practical use. Fourth, the optics were implemented as a benchtop prototype rather than a wearable device, and a head-mounted realization will require additional engineering considerations, including miniaturization of the light source and computing platform, thermal management, and power efficiency for extended wear. Finally, the present design uses laser sources at 473 and 660 nm; extension to a wider range of CVD severities and to deuteranopia-specific assistance will require further optimization of the recording wavelengths and Bragg-matched bands. Addressing these limitations is identified as essential future work to translate the present proof-of-concept into a clinically and practically deployable color-vision assistance system.

## Resource availability

### Lead contact

Requests for further information and resources should be directed to and will be fulfilled by the lead contact, Young-Wan Choi (ychoi@cau.ac.kr).

### Materials availability

This study did not generate any unique reagents.

### Data and code availability

• All data reported in this study will be shared by the lead contact upon request.
• All original codes used in this study are publicly available on GitHub (https://github.com/qldthe8675/Color-extraction).
• Any additional information required to reanalyze the data reported in this study is available from the lead contact upon request.

## Acknowledgments

This research was supported by the 10.13039/501100002460Chung-Ang University Graduate Research Scholarship in 2024, National Research Foundation (NRF) grants 10.13039/501100014188Ministry of Science and ICT (RS-2023-NR076420), and 10.13039/501100003661Korea Institute for Advancement of Technology (P0017011).

## Author contributions

S.-H.C. realized the proposed method and discussed the results. D.-H.B. performed experiments with the waveguide display and conducted measurements of the dual-color-based diffractive optics. W.J.C. simulated the CVD perception and drafted the manuscript. Y.-W.C. conceived the idea and supervised the project. All authors discussed the results and reviewed the manuscript.

## Declaration of interests

The authors declare no competing interests.

## STAR★Methods

### Key resources table

**Table:** 

REAGENT or RESOURCE	SOURCE	IDENTIFIER
**Software and algorithms**		

MATLAB R2022b	MathWorks	https://www.mathworks.com
Ansys Zemax OpticStudio 2023 R2.00	Ansys	https://www.ansys.com
Visual Studio Code 1.101.1	Microsoft	https://www.microsoft.com
Python 3.13.3	Python	https://www.python.org
OpenCV	Open source	N/A
Pandas	Open source	N/A
NumPy	Open source	N/A
Color	Open source	N/A
Matplotlib	Open source	N/A
SciPy	Open source	N/A
Visio 2013	Microsoft	https://www.microsoft.com
Origin 2018 64bit	OriginLab	https://www.originlab.com

**Other**		

Bayfol® HX200 photopolymer	Covestro Co., Ltd.	https://www.covestro.com
473 nm, 660 nm Cobolt 05-01 Series laser	HÜBNER Photonics Co., Ltd.	https://hubner-photonics.com
Fiber-coupled tungsten-halogen light source	Thorlabs Co., Ltd.	https://www.thorlabs.com
CCD spectrometer	Thorlabs Co., Ltd.	https://www.thorlabs.com
Beam projector	Epson Co., Ltd.	https://www.epson.com
CCD camera	Teledyne Co., Ltd.	https://www.teledynevisionsolutions.com/
Optical components	Thorlabs Co., Ltd.	https://www.thorlabs.com
ChromaGen lenses	ChromaGen Co., Ltd.	https://www.chromagen.us

### Experimental model and study participant details

This study does not involve an experimental model or subject in the life sciences.

### Method details

#### Fabrication of the CM-HOE

The CM-HOE in this manuscript was implemented using the optical interferometer that we built ourselves. The CM-HOE was recorded in a Bayfol HX200 photopolymer film (Geola; nominal thickness 16 μm), attached to a transparent soda-lime slide glass (26 × 76 × 1 mm) that subsequently served as the waveguide. Two diode-pumped solid-state lasers (Cobolt 05-01 series, HÜBNER Photonics) with center wavelengths of 473 nm and 660 nm were combined co-linearly through dichroic mirrors, expanded by a 20× beam expander (GBE20-A, Thorlabs), and split into signal and reference arms with a 50:50 polarizing beam splitter. The P-polarized arm was converted to S-polarization by a half-wave plate so that both recording beams were S-polarized. This ensured a high-contrast interference pattern within the photopolymer. The two S-polarized beams were directed at a relative half-angle of 60° into a 60° triangular prism (43–649, Edmund Optics), which was optically contacted to the photopolymer film via glycerin (*n* = 1.44) for index matching. The exposed area was 15 × 15 mm. For each recording wavelength, the signal- and reference-beam intensities were matched to within ±5% (measured with a PM100D power meter, Thorlabs) to maximize fringe visibility. The 473 nm and 660 nm beams were sequentially exposed in segments until a total energy of 30 mJ/cm^2^ was reached. After recording, the glycerin was fully removed with lens tissue, and each sample was cured under a broadband UV lamp for 3 min to stabilize the refractive-index modulation and prevent post-exposure drift. Two such samples were then bonded to opposite ends of a common slide-glass to form the input and output couplers of the waveguide display (Figure 2B).

#### Transmission spectrum measurement of CM-HOE

Transmission spectra of the six CM-HOE samples were acquired on the optical bench shown in Figure 3A. A fiber-coupled tungsten–halogen lamp (OSL2, Thorlabs) served as the broadband source. The output beam was collimated by a plano-convex lens and incident normally onto each sample. The transmitted spectrum *I*_*t*_*(λ)* was captured in a wavelength range of 400–700 nm by a CCD spectrometer (CCS100/M, Thorlabs) with 2 nm spectral resolution. Each sample was measured five times (independent alignments, *n* = 5 per sample). The mean spectrum was used for downstream analysis.

#### Ray tracing simulation of the CM-HOE-based waveguide display

Ray tracing simulation of the CM-HOE-based waveguide display (Figure 4A) was performed in Ansys Zemax OpticStudio. The geometry comprised a planar glass waveguide (*n* = 1.52, thickness 1 mm) with two reflection-type HOEs at its ends. Each coupler was specified with its Bragg-matched center wavelengths and incidence angles derived from the recording geometry. Input rays were launched at normal incidence to the input coupler. Only the R and B rays satisfied the Bragg condition and were diffracted into the TIR angle, whereas the G rays were transmitted off-Bragg without coupling into the waveguide. Relative diffraction-efficiency spectra (Figure 4B) were calculated with Kogelnik’s coupled-wave theory in MATLAB R2022b using photopolymer parameters consistent with the recording conditions, such as index modulation *Δn* = 0.03, grating thickness *d* = 16 μm, and a 2% post-cure shrinkage factor.

#### CVD perception simulation

Simulated CVD percepts (Figures 1C and 4C) were generated in MATLAB R2022b using the two-step pipeline of Viénot. Input RGB images were first converted to the LMS cone-excitation space via the matrix *M*_RGB→LMS_ given in the manuscript. The protanopia matrix *M*_protanomaly_ was then applied to project the trichromatic LMS values onto the dichromatic subspace. The simulation result was inverse transformed back to sRGB for display. Each pipeline was validated against reference simulations from the Machado et al. physiologically based model to confirm consistency.

#### CIE 1976 u’v' chromaticity and Δu’v' computation

Chromaticity coordinates were computed from the measured input and eyebox-output spectra (Figures 5A and 5B) using the CIE 1931 2° observer color-matching functions. The tristimulus values *X*, *Y*, *Z* were calculated by numerical integration (Simpson’s rule, 1 nm step) of the spectrum product and the CIE 1931 2° color-matching functions. The (*x*, *y*) chromaticity coordinates were converted to (*u′*, *v′*) via the standard CIE 1976 transforms given in Equations 6 and 7 of the manuscript. The perpendicular distance Δ*u′v′* from the protanopic confusion line was calculated using the standard protanopic copunctal point (*u*_*p*_, *v*_*p*_) = (0.658, 0.501) from the CIE 1931 2° observer.

#### Benchtop proof-of-concept AR experiment

The benchtop system (Figure 7A) comprised an Epson EF-11 beam projector as the image source, a plano-convex collimating lens (f = 50 mm), the CM-HOE-based waveguide display, a Logitech C920 Pro webcam (resolution 1080p) fixed along the waveguide’s optical axis for real-time color tracking, and a CCD camera positioned 15 mm from the output coupler to emulate the typical eye-relief distance of a pair of eyeglasses. The webcam output was streamed into the ANN for real-time R color region detection. Binary masks identifying R color regions were transmitted to the projector, which then illuminated only those regions via the waveguide. Captured images were post-processed with the CVD simulation described above to show the predicted appearance to a protanomalous observer. End-to-end latency (webcam capture, ANN inference, projector display) was measured on a laptop with an Intel Core i5-10210U CPU.

#### ANN for HSV-based red color object extraction

A feedforward ANN-based color tracking algorithm was implemented in Python 3.13.3 with OpenCV, Pandas, NumPy, Color, Matplotlib, and SciPy in Visual Studio Code 1.101.1. The network architecture (Figure 6A) consisted of a three-node input layer (H, S, V channels after RGB→HSV conversion), two fully connected hidden layers with 20 and 10 nodes, respectively, and a single-node binary output indicating the presence or absence of R. A symmetric sigmoid activation function was used for hidden-layer activations. The training dataset consisted of 1,000 annotated images of R and non-R objects captured under varied illumination conditions (indoor/outdoor, low-light, and shadow-dominant scenes). Images were randomly partitioned into training (70%, *n* = 700), validation (15%, *n* = 150), and held-out test (15%, *n* = 150) subsets. Partitioning used a fixed random seed for reproducibility. Training was stopped once validation loss saturated, which occurred near epoch 100. Model robustness was probed by the fast gradient sign method (FGSM). Perturbations were applied at ε = 0.1 and ε = 0.2 on the full test set. Masked outputs are shown in Figure 6C.

#### FoV, eyebox, and modulation transfer function characterization

The FoV of our waveguide display was measured by scanning the projector over a range of input angles and recording the angle beyond which the diffracted intensity at the eyebox fell below 50% of its peak value. The eyebox was measured by translating the CCD camera laterally at the nominal 15 mm eye relief distance and recording the area over which image intensity remained above 50% of peak. The modulation transfer function (MTF) was evaluated using the slanted-edge method with a projected image via the waveguide display. The MTF value reported in the manuscript is the contrast at the highest resolvable spatial frequency.

### Quantification and statistical analysis

All quantitative analyses were performed in MATLAB R2022b (MathWorks) and Python 3.13.3 with an Intel Core i5 CPU. The ANN-based color tracking algorithm and CVD vision simulation were realized in Python 3.13.3. As this proof-of-concept study characterizes the optical performance of a device rather than comparing experimental groups, no inferential statistical tests (t-tests, ANOVA, *p* values) were performed. Descriptive measurements with their sample sizes are summarized in this section. Relative diffraction efficiencies were measured for *n* = 6 fabricated CM-HOE samples (Figure 3B), each measured under five independent alignments (*n* = 5 per sample). Peak efficiencies ranged from 11 to 40% at 473 nm and 36–57% at 660 nm. Bragg-matched diffraction was observed over 464–479 nm and 651–682 nm. Chromaticity coordinates were computed in the CIE 1976 u′v′ color space using the CIE 1931 2° observer color-matching functions and the standard protanopic copunctal point (*u*_*p*_, *v*_*p*_) = (0.658, 0.501). The measured input chromaticity was (u_i_′, v_i_′) = (0.23798, 0.52864) and eyebox-output chromaticity was (*u′*, *v′*) = (0.31896, 0.49616), yielding *Δu′v′* = 0.02709 in Figure 5. Accuracy, precision, and recall were computed on the held-out test set (*n* = 150 images) following data partitioning of 70% (*n* = 700) training/15% (*n* = 150) validation/15% (*n* = 150) test in Figure 6B. Training was stopped after ∼100 epochs upon validation-loss saturation. FGSM robustness was evaluated at ε = 0.1 and ε = 0.2 on the full test set in Figure 6C. End-to-end latency was measured over *n* = 20 trials on a laptop with an Intel Core i5-10210U CPU. Latency ranged 38.5–52.7 ms across measurements. FoV was 4.4°, eyebox 42 mm^2^, and MTF 0.5 at a spatial frequency of 14.2 lp/mm, all measured on the prototype shown in Figure 7A.
